# The association between the clinical severity of heart failure and docosahexaenoic acid accumulation in hypertrophic cardiomyopathy

**DOI:** 10.1186/s13104-022-06023-1

**Published:** 2022-04-14

**Authors:** Keitaro Akita, Kenji Kikushima, Takenori Ikoma, Ariful Islam, Tomohito Sato, Taisei Yamamoto, Tomoaki Kahyo, Mitsutoshi Setou, Yuichiro Maekawa

**Affiliations:** 1 Division of Cardiology, Internal Medicine III, Hamamatsu University School of Medicine, 1-20-1 Handayama, Higashi-ku, Hamamatsu, 431-3192 Japan; 2 Department of Cellular and Molecular Anatomy and International Mass Imaging Center, Hamamatsu University School of Medicine, 1-20-1 Handayama, Higashi-ku, Hamamatsu, 431-3192 Japan

**Keywords:** Hypertrophic cardiomyopathy, Endomyocardial biopsy, Imaging mass spectrometry, Docosahexaenoic acid

## Abstract

**Objective:**

Hypertrophic cardiomyopathy (HCM) is a common genetic disease with diverse morphology, symptoms, and prognosis. Hypertrophied myocardium metabolism has not been explored in detail. We assessed the association between myocardium lipid metabolism and clinical severity of heart failure (HF) in HCM using imaging mass spectrometry (IMS).

**Results:**

We studied 16 endomyocardial biopsy (EMB) specimens from patients with HCM. Analysis was conducted using desorption electrospray ionization IMS. The samples were assigned into two cohorts according to the period of heart biopsy (cohort 1, n = 9 and cohort 2, n = 7). In each cohort, samples were divided into two groups according to the clinical severity of HF in HCM: clinically severe and clinically mild groups. Signals showing a significant difference between the two groups were analyzed by volcano plot. In cohort 1, the volcano plot identified four signals; the intensity in the clinically severe group was more than twice that of the mild group. Out of the four signals, docosahexaenoic acid (DHA) showed significant differences in intensity between the two groups in cohort 2 (10,575.8 ± 2750.3 vs. 19,839.3 ± 4803.2, *P* = 0.025). The intensity of DHA was significantly higher in EMB samples from the clinically severe HCM group than in those from the mild group.

## Introduction

Hypertrophic cardiomyopathy (HCM) is the most common genetic cardiomyopathy, with a prevalence of approximately 0.2% worldwide [1]. The clinical characteristics, myocardial morphology, symptoms, and prognosis of patients with HCM are highly diverse. Heart failure (HF) is one of the major causes of death in patients with HCM, and it is challenging to prevent the onset of HF in these patients.

In terms of cell metabolism, hypertrophied myocardium requires a large amount of energy, and often presents with relative myocardial ischemia, irrespective of the presence of gene mutations [2, 3]. In HCM with HF, metabolic disproportion is likely to occur [4]. This abnormal energy metabolism in HCM has long been clinically confirmed by positron emission tomography-computed tomography as a hyperglycemic state. In recent years, imaging mass spectrometry (IMS) has made it possible to analyze energy metabolism at the cellular level in detail and in a shorter time. Although the intramyocardial lipid metabolism in patients with advanced HF has been described [5], the details regarding the differences in fatty acid (FA) metabolism in the hypertrophied myocardium of HCM according to the severity of HF are yet to be elucidated. Endomyocardial biopsy (EMB) is not only essential for monitoring rejection of transplanted hearts but is also useful in differentiating primary cardiomyopathy from myocarditis and other specific secondary cardiomyopathies, and sometimes in diagnosing cardiac tumors. As far as we know, there are no reports of myocardial metabolism analyzed using EMB samples. IMS enables the prompt assessment of energy metabolism, including FA metabolism in the myocardium using EMB samples. The purpose of this study was to explore the details of FA metabolism from HCM cases using IMS.

## Main text

### Methods

#### Patients

A total of 16 patients with HCM were enrolled at Hamamatsu University Hospital from December 2018 to June 2020, and 16 endomyocardial biopsy specimens were obtained from each patient. We divided the samples into two groups, namely the clinically severe group (eight patients) and clinically mild group (eight patients) according to their necessity of invasive therapy including implantable cardioverter defibrillator (ICD) or septal reduction therapy (SRT). Indication for ICD was determined by the calculated risk of sudden cardiac death (SCD) within 5 years, using the HCM-SCD risk calculator [6]. Indications for SRT were determined based on the ACC/AHA and ESC guidelines [7, 8]. One patient who clinically required ICD but declined was assigned to the severe group. The participants provided written consent before the study procedures began. The institutional review board of Hamamatsu University School of Medicine approved all aspects of this study (application number 17-261). The study was conducted in accordance with the regulations of Declaration of Helsinki.

#### Endomyocardial biopsy

EMBs were performed in a cardiac catheterization laboratory by a cardiology consultant with expertise in the procedure. EMB were most commonly performed through a 9 French right femoral venous access sheath with the use of a long catheter and a bioptome. A minimum of three specimens were obtained from the right ventricular septum. Left ventricular EMB was not performed in any of the cases.

#### DESI-IMS

Heart biopsy samples were immediately frozen on dry ice, prepared freeze block using the Super Cryoembedding Medium and stored at − 80 °C until sectioning. All the specimens were sectioned at 10 μm thickness using a cryostat (CM1950, Leica Biosystems, Wetzlar, Germany) at − 20 °C on glass slides. Heart biopsy sections were kept at room temperature for a while to dry just before IMS acquisition.

All the experiments were performed using a desorption electrospray ionization (DESI) source attached to a quadrupole time-of-flight mass spectrometer (Xevo G2-XS Q-TOF, Waters, Milford, MA, USA). The DESI-IMS mass spectra were calibrated externally using a 500 µM sodium formate solution in 90% 2-propanol, prior to measurement. To obtain the maximum signal intensity from heart biopsy samples, DESI parameters were optimized as previously described [9] with slight modifications. All the candidate molecules corresponding to each targeted *m/z* were selected using the Human Metabolome Database (https://hmdb.ca/) based on their mass accuracy and biological distribution.

#### Data analysis

The first cohort analysis was performed on the severe (n = 4) and the mild (n = 5) group samples. The data were evaluated using a single blind method; only the researchers were informed of the assignments of each sample, but they were blinded to the clinical severity of the samples. The signal intensities from the sample area on the IMS image of each sample were selected, and the difference in the mass spectra between the two groups was analyzed using the volcano plot (Microsoft Excel version 2019, Microsoft Corp, Redmond, WA, USA). The longitudinal axis represents the log_2_ of fold changes of the mass intensities of the same *m/z* between the two groups, and the vertical axis represents − log_10_ of the *P* value obtained by the *t*-test between the two groups. We selected the signals with both *P* < 0.05 and fold change of ≥ 2, between the severe and mild groups. From the IMS images of these candidates, we confirmed that these signals were obtained from the sample area and were not background noises.

#### Examination of the candidate validity and signal identification

The validity of the obtained signals was confirmed using a cohort study. The seven samples of cohort 2 were divided into the severe (n = 4) and mild (n = 3) groups. The signal intensity and distribution of the candidates identified in cohort 1 were examined in cohort 2 samples. The variation of m/z in cohort 1 and 2 measurements was corrected by other common molecules with peaks near the candidate’s m/z. Differences with *P* < 0.05 were considered significant and signals with *P* < 0.05, were selected as candidates.

#### Statistical analysis

For patient characteristics, continuous variables are expressed as mean ± standard deviation, and categorical data as absolute values and percentages. Independent continuous variables were compared using Student’s t tests, and categorical variables with Pearson’s chi square test. All the *P* values were two-sided. Results were considered statistically significant at a *P* < 0.05. Analyses were performed using IBM SPSS Statistics version 26 (IBM, Armonk, New York, USA).

## Results

**Fig. 1:**
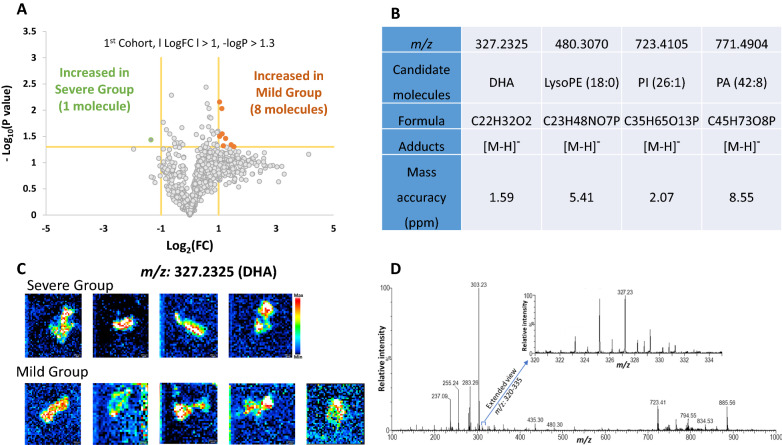
Signal intensity and volcano plot of heart biopsy samples from cohort 1. **A** The volcano plot of cohort 1 samples was divided into two groups according to the clinical severity. **B** Out of the nine molecules that significantly increased in either group, four molecules were localized in the biopsy samples as observed by DESI-IMS imaging. **C** DESI-IMS imaging of DHA in each biopsy sample from cohort 1. **D** Representative mass spectrum of DHA from cohort 1. *DESI-IMS* desorption electrospray ionization imaging mass spectrometry; *DHA* docosahexaenoic acid; *LysoPE* lysophosphatidylethanolamine; *PI* phosphatidylinositol; *PA* phosphatidic acid

**Fig. 2:**
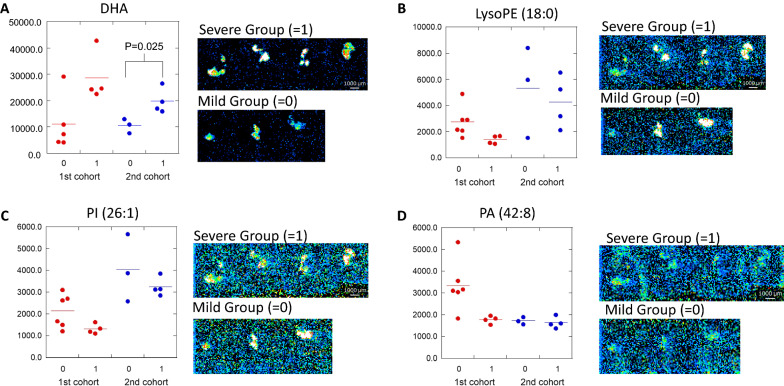
Comparison of signal intensities of candidate molecules between the clinically mild and severe groups in each cohort. **A** DHA, **B** Lyso PE, **C** PI, **D** PA. **A**–**D** The left side is the signal intensity plots of each molecule (cohort 1: red plots, 2nd cohort 2: blue plots). In each plot, “0” is the clinically mild group, and “1” is the clinically severe group. The right side shows a DESI-IMS image of each molecule from each biopsy sample of cohort 2. *DESI-IMS* desorption electrospray ionization imaging mass spectrometry; *DHA* docosahexaenoic acid; *LysoPE* lysophosphatidylethanolamine; *PI* phosphatidylinositol; *PA* phosphatidic acid

The patient’s baseline characteristics are shown in Table 1. Patients in the severe group had a higher NYHA class, higher history of unexplained syncope, higher risk of sudden cardiac death in 5 years according to the HCM-SCD risk calculator, and greater left ventricular mass in cardiac magnetic resonance imaging compared with those in the mild group. From the volcano plot of cohort 1 samples, nine signals were identified [*P* < 0.05 and |log_10_(fold change) |> 1] (Fig. 1A). Among them, four molecules were localized in the biopsy samples in DESI-IMS, including docosahexaenoic acid (DHA), lysophosphatidylethanolamine (18:0), phosphatidylinositol (26:1), and phosphatidic acid (42:8) (Fig. 1B). The image and mass spectrum of DHA were shown as representative data in Fig. 1C and D, respectively. We could confirm the signals of these four candidate molecules from cohort 2 samples (severe group, four patients; mild group, three patients). We plotted and compared the signal intensities of the candidate molecules between the severe and mild groups. In both cohorts 1 and 2, the intensities of DHA of the severe groups were significantly higher than those of the mild groups (Fig. 2A). There were no significant differences in the intensities of the other three molecules between the two groups (Fig. 2B–D). There were no sex differences in the intensities of DHA (female, 15,753.2 ± 13,547.3; male, 19,008.9 ± 7933.6, *P* = 0.56).

**Table 1:** Baseline clinical characteristics of the study population

	Mild (N = 8)	Severe (N = 8)	*P*
Age (years)	70.5 ± 11.6	56.4 ± 21.8	0.13
Weight (kg)	61.4 ± 11.9	59.0 ± 16.2	0.75
BMI (kg/m^2^)	25.0 ± 5.0	22.6 ± 4.5	0.34
Female, N (%)	5 (62.5)	3 (37.5)	0.32
NYHA class			**0.029**
I	3 (37.5)	0 (0.0)	
II	5 (62.5)	4 (50.0)	
III	0 (0.0)	4 (50.0)	
IV	0 (0.0)	0 (0.0)	
History of cardiopulmonary arrest, N (%)	0 (0.0)	0 (0.0)	
History of unexplained syncope, N (%)	1 (12.5)	5 (62.5)	**0.039**
Family history of sudden cardiac death, N (%)	0 (0.0)	1 (12.5)	0.3
Documentation of NSVT, N (%)	1 (12.5)	2 (25.0)	0.5
Risk of sudden cardiac death in 5 years (%)	1.58 ± 1.08	4.31 ± 2.93	**0.036**
Atrial fibrillation, N (%)	3 (37.5)	1 (12.5)	0.25
Hypertension, N (%)	4 (50.0)	5 (62.5)	0.61
Diabetes, N (%)	1 (12.5)	1 (12.5)	1.0
Dyslipidemia, N (%)	3 (37.5)	2 (25.0)	0.59
Current smoker, N (%)	2 (25.0)	1 (12.5)	0.52
COPD, N (%)	1 (12.5)	0 (0.0)	0.3
Coronary artery disease, N (%)	0 (0.0)	0 (0.0)	
Stroke, N (%)	0 (0.0)	0 (0.0)	
Malignancy, N (%)	1 (12.5)	1 (12.5)	1.0
Medication, N (%)			
Na channel blocker	1 (12.5)	2 (25.0)	0.52
Beta-blocker	5 (62.5)	6 (75.0)	0.59
Calcium channel blocker	5 (62.5)	2 (25.0)	0.13
ACE inhibitors or ARBs	3 (37.5)	4 (50.0)	0.61
Anticoagulation	2 (25.0)	1 (12.5)	0.52
Amiodarone	0 (0.0)	1 (12.5)	0.3
Metformin	1 (12.5)	0 (0.0)	0.3
Statin	1 (12.5)	1 (12.5)	1.0
Fibrate	0 (0.0)	1 (12.5)	0.3
Ezetimib	0 (0.0)	0 (0.0)	
EPA	0 (0.0)	0 (0.0)	
DHA	0 (0.0)	0 (0.0)	
Operations and interventions, N (%)			
Surgical myectomy	0 (0.0)	0 (0.0)	
Alcohol septal ablation	0 (0.0)	5 (62.5)	**0.007**
AF ablation	2 (25.0)	0 (0.0)	0.13
ICD implantation	0 (0.0)	2 (25.0)	0.13
Vital signs			
Systolic blood pressure (mmHg)	121.0 ± 11.7	121.4 ± 11.9	0.95
Diastolic blood pressure (mmHg)	73.8 ± 13.3	66.2 ± 7.4	0.19
Heart rate (bpm)	59.0 ± 8.4	66.1 ± 6.4	0.077
Laboratory data			
NT-proBNP (pg/mL)	477.4 ± 471.7	1482 ± 1497	0.092
Troponin T (ng/mL)	0.0129 ± 0.008	0.0254 ± 0.022	0.15
CRP (mg/dL)	0.13 ± 0.11	0.07 ± 0.04	0.24
Hemoglobin (g/dL)	12.7 ± 1.7	13.3 ± 2.4	0.60
Creatinine (mg/dL)	0.90 ± 0.21	0.83 ± 0.31	0.80
eGFR (mL/min/1.73 m^2^)	57.1 ± 15.6	65.8 ± 29.0	0.47
HbA1c (%)	5.9 ± 0.6	5.6 ± 0.4	0.34
LDL-C (mg/dL)	106.3 ± 22.8	99.1 ± 19.2	0.51
Dihomo-gamma-linolenic acid (µg/mL)	38.2 ± 12.0	41.2 ± 13.8	0.67
Arachidonic acid (AA) (µg/mL)	201.4 ± 33.0	197.0 ± 55.0	0.86
EPA (µg/mL)	73.4 ± 52.7	46.8 ± 20.5	0.21
DHA (µg/mL)	115.0 ± 46.9	127.3 ± 39.6	0.59
EPA/AA ratio	0.39 ± 0.28	0.24 ± 0.09	0.22
DHA/AA ratio	0.60 ± 0.32	0.66 ± 0.21	0.66
Echocardiographic variables			
Maximum LV wall thickness (mm)	17.3 ± 4.6	19.4 ± 2.4	0.28
LVEF (%)	72.0 ± 7.1	70.2 ± 4.7	0.56
LAD (mm)	41.0 ± 7.3	38.4 ± 2.5	0.37
LAVI (mL/m^2^)	53.5 ± 20.5	46.1 ± 5.5	0.39
E/A	0.73 ± 0.18	0.84 ± 0.53	0.58
E/e’	17.3 ± 5.2	16.4 ± 7.4	0.79
LVOT-PG (mmHg)	32.3 ± 43.4	59.8 ± 37.5	0.20
MRI variables			
LV mass (g)	112.5 ± 39.5	166.6 ± 40.4	**0.017**
Apical aneurysm, N (%)	1 (12.5)	2 (25.0)	0.52
LGE, N (%)	3 (37.5)	4 (50.0)	0.61

Bold values denote statistical significance at the *P* < 0.05.

Data are expressed as mean ± SD and number (%)

*BMI* body mass index; *NYHA* New York Heart Association; *NSVT* non-sustained ventricular tachycardia; *COPD* chronic obstructive pulmonary disease; *ACE* angiotensin-converting enzyme; *ARB* angiotensin receptor blocker; *EPA* eicosapentaenoic acid; *DHA* docosahexaenoic acid; *AF* atrial fibrillation; *ICD* implantable cardioverter defibrillator; *NT-proBNP* n-terminal pro-brain natriuretic peptide; *CRP* C-reactive protein; *eGFR* estimated glomerular filtration rate; *LV* left ventricle; *LAD* left atrial diameter; *LAVI* left atrial volume index; *LVOT-PG* left ventricular outflow tract-pressure gradient; *MRI* magnetic resonance imaging; *LGE* late gadolinium enhancement

## Discussion

In this study, we compared the myocardial metabolism between patients with clinically severe and mild HCM using DESI-IMS. We revealed that the myocardium in the clinically severe HCM group had significantly higher levels of DHA.

Our study is unique because it used the DESI-IMS to highlight the details of FA metabolic disorders. To date, the following studies have been conducted on this topic: one used resected myocardium from patients with severe HF in whom heart transplant was indicated [10] and the other used resected myocardium from patients with hypertrophic obstructive cardiomyopathy in whom septal myectomy was indicated [11]. However, only the severe cases were examined, and IMS was not used for analysis in both studies. Although EMB is an invasive procedure, it is a validated method for diagnosing cardiomyopathies [7, 12]. EMB can be performed irrespective of the presence of clinical symptoms and their degree of severity, to differentiate even the early or mild phase of other cardiomyopathies from HCM. Therefore, this study could include the mild cases. However, a non-invasive method will be required to accurately determine if DHA accumulates in the myocardium or not, especially in asymptomatic or mildly symptomatic patients with HCM.

Adenosine triphosphate (ATP) production in the myocardium mainly relies on the mitochondrial oxidation of FA, carbohydrates, ketone bodies, and amino acids. The remaining ATP is produced through aerobic glycolysis [3]. In normal hearts, FAs account for the majority of oxidative metabolism. However, in hypertrophied hearts, FA oxidation is reduced, and glucose utilization is increased [13]. Therefore, it can be assumed that FA pooling is enhanced in hypertrophied myocardium. Ranjbarvaziri et al. reported that free FA accumulation in HCM myocardium was significantly higher than that in normal myocardium [14]. Additionally, they stated that the dysregulation in FA metabolism in HCM myocardium may be caused by mitochondrial damage and reduced citrate synthase activity which was associated with increased reactive oxygen species, based on the findings from electron microscopy and integrated molecular pathway level analysis. This report supports the hypothesis that some free FAs, including DHA, are not properly metabolized, and are abnormally pooled in the hypertrophied myocardium.

However, the fact that the intensity of DHA was higher in the myocardium obtained from the severe HCM group shows that DHA could have some implications, rather than decreased FA oxidation in hypertrophied hearts.

DHA is an omega-3 polyunsaturated fatty acid (PUFA), that has been reported to have several beneficial effects on the human body, such as anti-inflammatory and anti-atherosclerotic effects. Omega-3 PUFAs have been reported to have anti-remodeling and anti-fibrotic effects on the myocardium in mice [15]. DHA in particular has been reported to accumulate in cardiac tissues compared to other omega-3 PUFAs (regardless of supplementation). DHA rather than EPA supplementation reduces vulnerability to atrial fibrillation [16]. In this study, the signal intensity of DHA in the clinically severe HCM group was significantly higher than that of the clinically mild HCM group, even though all the patients did not receive DHA supplementation. Previous studies reported DHA to have an inhibiting effect on phenylephrine-induced cardiac hypertrophy under in-vitro situations [17, 18]. However, another report from an in-vivo animal investigation documented the serum DHA concentration in HCM was not higher than the control [19]. Integrating these results, DHA itself has cardioprotective effects; however, its utilization might be dysregulated under the condition of severe HCM with mitochondrial damage.

Although the precise mechanism of upregulation of DHA in severe HCM myocardium is unknown, we could hypothesize that higher oxidative stress in severely hypertrophied myocardium induces the accumulation of DHA.

From the analysis using DESI-IMS, the intensity of DHA was significantly higher in the EMB samples from the clinically severe HCM group than in those from the mild HCM group. DHA may play an important role in the pathophysiology of worsening HF in patients with HCM.

## Limitations

To prove this hypothesis, further research is needed to reveal the cascade that is upgrading these FAs in severe HCM myocardium. Verification studies with larger samples sizes are also required. However, this study suggests the possibility of identifying the metabolism of patients with severe HCM using a novel method and reveals the pathophysiology of worsening HF in HCM hearts.

## Data Availability

Participants in this study did not agree to the public sharing of their data so supporting data is not available.

## Abbreviations

HCM: Hypertrophic cardiomyopathy
HF: Heart failure
IMS: Imaging mass spectrometry
EMB: Endomyocardial biopsy
DHA: Docosahexaenoic acid
FA: Fatty acid
ICD: Implantable cardioverter defibrillator
SRT: Septal reduction therapy
SCD: Sudden cardiac death
DESI: Desorption electrospray ionization
ATP: Adenosine triphosphate
PUFA: Polyunsaturated fatty acid

## References

[1] Maron BJ, Mathenge R, Casey SA, Poliac LC, Longe TF (1999). Clinical profile of hypertrophic cardiomyopathy identified de novo in rural communities. J Am Coll Cardiol.

[2] Ritterhoff J, Tian R (2017). Metabolism in cardiomyopathy: every substrate matters. Cardiovasc Res.

[3] Greenwell AA, Gopal K, Ussher JR (2020). Myocardial energy metabolism in non-ischemic cardiomyopathy. Front Physiol.

[4] Vakrou S, Abraham MR (2014). Hypertrophic cardiomyopathy: a heart in need of an energy bar?. Front Physiol.

[5] Bedi KC, Snyder NW, Brandimarto J, Aziz M, Mesaros C, Worth AJ (2016). Evidence for intramyocardial disruption of lipid metabolism and increased myocardial ketone utilization in advanced human heart failure. Circulation.

[6] O'Mahony C, Jichi F, Pavlou M, Monserrat L, Anastasakis A, Rapezzi C (2014). A novel clinical risk prediction model for sudden cardiac death in hypertrophic cardiomyopathy (HCM risk-SCD). Eur Heart J.

[7] Elliott PM, Anastasakis A, Borger MA, Borggrefe M, Cecchi F, Authors/Task Force Members (2014). 2014 ESC Guidelines on diagnosis and management of hypertrophic cardiomyopathy: the Task Force for the Diagnosis and Management of Hypertrophic Cardiomyopathy of the European Society of Cardiology (ESC). Eur Heart J.

[8] Ommen SR, Mital S, Burke MA, Day SM, Deswal A, Elliott P (2020). 2020 AHA/ACC guideline for the diagnosis and treatment of patients with hypertrophic cardiomyopathy: a report of the American College of Cardiology/American Heart Association Joint Committee on Clinical Practice Guidelines. J Am Coll Cardiol.

[9] Islam A, Takeyama E, Mamun MA, Sato T, Horikawa M, Takahashi Y (2020). Green nut oil or DHA supplementation restored decreased distribution levels of DHA containing phosphatidylcholines in the brain of a mouse model of dementia. Metabolites.

[10] Diakos NA, Selzman CH, Sachse FB, Stehlik J, Kfoury AG, Wever-Pinzon O (2014). Myocardial atrophy and chronic mechanical unloading of the failing human heart: implications for cardiac assist device-induced myocardial recovery. J Am Coll Cardiol.

[11] Guo Y, Wu X, Zheng X, Lu J, Wang S, Huang X (2017). Usefulness of preoperative transforming growth factor-beta to predict new onset atrial fibrillation after surgical ventricular septal myectomy in patients with obstructive hypertrophic cardiomyopathy. Am J Cardiol.

[12] Kitaoka H, Tsutsui H, Kubo T, Ide T, Chikamori T, Fukuda K (2021). JCS/JHFS 2018 guideline on the diagnosis and treatment of cardiomyopathies. Circ J.

[13] Stanley WC, Recchia FA, Lopaschuk GD (2005). Myocardial substrate metabolism in the normal and failing heart. Physiol Rev.

[14] Ranjbarvaziri S, Kooiker KB, Ellenberger M, Fajardo G, Zhao M, Vander Roest AS (2021). Altered cardiac energetics and mitochondrial dysfunction in hypertrophic cardiomyopathy. Circulation.

[15] Toko H, Morita H, Katakura M, Hashimoto M, Ko T, Bujo S (2020). Omega-3 fatty acid prevents the development of heart failure by changing fatty acid composition in the heart. Sci Rep.

[16] Ramadeen A, Connelly KA, Leong-Poi H, Hu X, Fujii H, Laurent G (2012). Docosahexaenoic acid, but not eicosapentaenoic acid, supplementation reduces vulnerability to atrial fibrillation. Circ Arrhythm Electrophysiol.

[17] Siddiqui RA, Shaikh SR, Kovacs R, Stillwell W, Zaloga G (2004). Inhibition of phenylephrine-induced cardiac hypertrophy by docosahexaenoic acid. J Cell Biochem.

[18] Castillo A, Ruzmetov N, Harvey KA, Stillwell W, Zaloga GP, Siddiqui RA (2005). Docosahexaenoic acid inhibits protein kinase C translocation/activation and cardiac hypertrophy in rat cardiomyocytes. J Mol Genet Med.

[19] Hall DJ, Freeman LM, Rush JE, Cunningham SM (2014). Comparison of serum fatty acid concentrations in cats with hypertrophic cardiomyopathy and healthy controls. J Feline Med Surg.

